# Case Reports of Pembrolizumab-induced Acute Inflammatory Demyelinating Polyneuropathy

**DOI:** 10.7759/cureus.3371

**Published:** 2018-09-27

**Authors:** Rupesh Manam, Jasmine L Martin, Joshua A Gross, Dhishna Chaudhary, Sajeel Chowdhary, Patricio S Espinosa, Edgardo S Santos

**Affiliations:** 1 Internal Medicine, Florida Atlantic University Charles E. Schmidt College of Medicine, Boca Raton, USA; 2 Neuro-Oncology, Marcus Neuroscience Institute, Boca Raton, USA; 3 Neurology, Marcus Neuroscience Institute, Boca Raton, USA; 4 Oncology, Lynn Cancer Institute, Boca Raton, USA

**Keywords:** pembrolizumab, acute inflammatory demyelinating polyneuropathy, aidp, guillain-barre syndrome, checkpoint inhibitor, programmed cell death 1 pathway, pd-1, humanized monoclonal antibody, immune-related adverse events

## Abstract

Pembrolizumab is a humanized monoclonal antibody that blocks the programmed cell death 1 (PD-1) pathway, thereby enhancing antitumor immunity. As the use of immune checkpoint inhibitors becomes more prevalent, so do immune-related adverse events associated with their use. The immune-related adverse events linked with this class of drugs are commonly seen and most of the time are classified as mild adverse events. These are easily treated with steroids if recognition of symptomatology and treatment are promptly established. However, neurologic immune-related adverse events are less understood and have been infrequently cited in the medical literature, thus representing a challenge for clinicians.

## Introduction

Pembrolizumab is a humanized monoclonal immunoglobulin (IgG4) antibody that binds to the program cell death 1 (PD-1) receptor and prevents interaction with its ligands program death-ligand 1 (PD-L1) and program death-ligand 2 (PD-L2). Somatic cells produce these ligand proteins that attach to the PD-1 receptor and induce inhibitory signals, which cause T-cell deactivation [1-2]. This process negatively regulates the immune system to prevent autoimmunity, thus the PD-1 receptor is recognized as an immune checkpoint. Tumor cells often upregulate PD-L1 and PD-L2 ligands on their cell surfaces to evade T-cell recognition. Pembrolizumab blocks this interaction, thereby preserving the T-cell mediated immune response, which ultimately leads to apoptosis of tumor cells [2-3]. 

PD-L1 and PD-L2 expressions have been found in many tumors, including melanoma, ovarian, lung, and renal cell carcinomas. Today, the clinical use of immune-oncology (IO) agents, such as checkpoint inhibitors, has significantly increased in many tumor types and clinical scenarios. Clinicians must be aware of all potential immune-related adverse events (irAEs) that could happen to an individual under the IO approach. Pembrolizumab has been granted several clinical approvals by the United States Food and Drug Administration for multiple malignancies, including metastatic melanoma, non-small cell lung cancer, kidney, bladder, head and neck cancers, and solid tumors, which have microsatellite instability. Herein, we present two cases of patients treated with pembrolizumab who subsequently developed progressive generalized weakness and areflexia within three to four weeks of treatment, consistent with acute inflammatory demyelinating polyneuropathy (AIDP). AIDP as an irAE, not only for pembrolizumab but also for all checkpoint inhibitors in general, has been infrequently described in the medical literature [4-10]. It is critical to immediately recognize this complication, as therapy must be permanently discontinued and treatment must be promptly delivered. Our aim is to better understand the neurologic complications and the current recommended treatment modalities for these neurological events.

## Case presentation

Case report 1

A 73-year-old Caucasian male with a biopsy-proven diagnosis of stage IV poorly differentiated adenocarcinoma of the lung, epidermal growth factor receptor mutation negative, anaplastic lymphoma kinase translocation negative, and PD-L1 tumor proportion score of 20% was started on carboplatin, pemetrexed, and pembrolizumab. The patient was receiving the chemotherapy regimen every three weeks and prior to initiating cycle two, he developed generalized weakness. On presentation, he expressed subjective progressive weakness of the lower extremities (LEs) greater than the upper extremities (UEs). The physical exam was significant for 3/5 motor strength and absent deep tendon reflexes in the bilateral upper extremities (UEs) and LEs. Given the clinical presentation, an irAE secondary to an IO agent was suspected, with a differential diagnosis that included Guillain-Barré syndrome (GBS) versus myasthenia gravis-like syndrome. Lumbar puncture revealed albuminocytological dissociation in the cerebrospinal fluid (CSF) of 68 g/L, which further supported AIDP. The paraneoplastic panel was negative. Infectious workup, including CSF cultures, cytogenetic studies, polymerase chain reaction (PCR) for herpes simplex virus, cytomegalovirus, and serum venereal disease research laboratory (VDRL) was also negative. Methylprednisolone, along with intravenous immunoglobulin (IVIG), were initiated. Despite five infusions of IVIG, the patient’s strength diminished to 2/5 in the bilateral UEs and LEs. IVIG treatments were stopped and plasmapheresis was initiated. On Day 8 of hospitalization, the patient was transferred to the intensive care unit (ICU) for worsening respiratory status, as indicated by the measured lowest negative inspiratory force (NIF) of -20 cm H2O and forced vital capacity (FVC) of 1.1 L. Fortunately, the patient’s respiratory status improved with plasmapheresis and high-dose corticosteroid treatments and he never required mechanical ventilation. The patient received a total of eight sessions of plasmapheresis with a gradual recovery of his motor function. At Day 25 of hospitalization, the patient’s strength in his right UE was 5/5, left UE 3/5, and bilateral LEs 3/5. He was discharged to an acute rehabilitation facility on low-dose prednisone with instructions to follow up in the outpatient department. Unfortunately, the patient was re-admitted one month after discharge with another irAE and was subsequently placed in hospice care.

Case report 2

**Figure 1:**
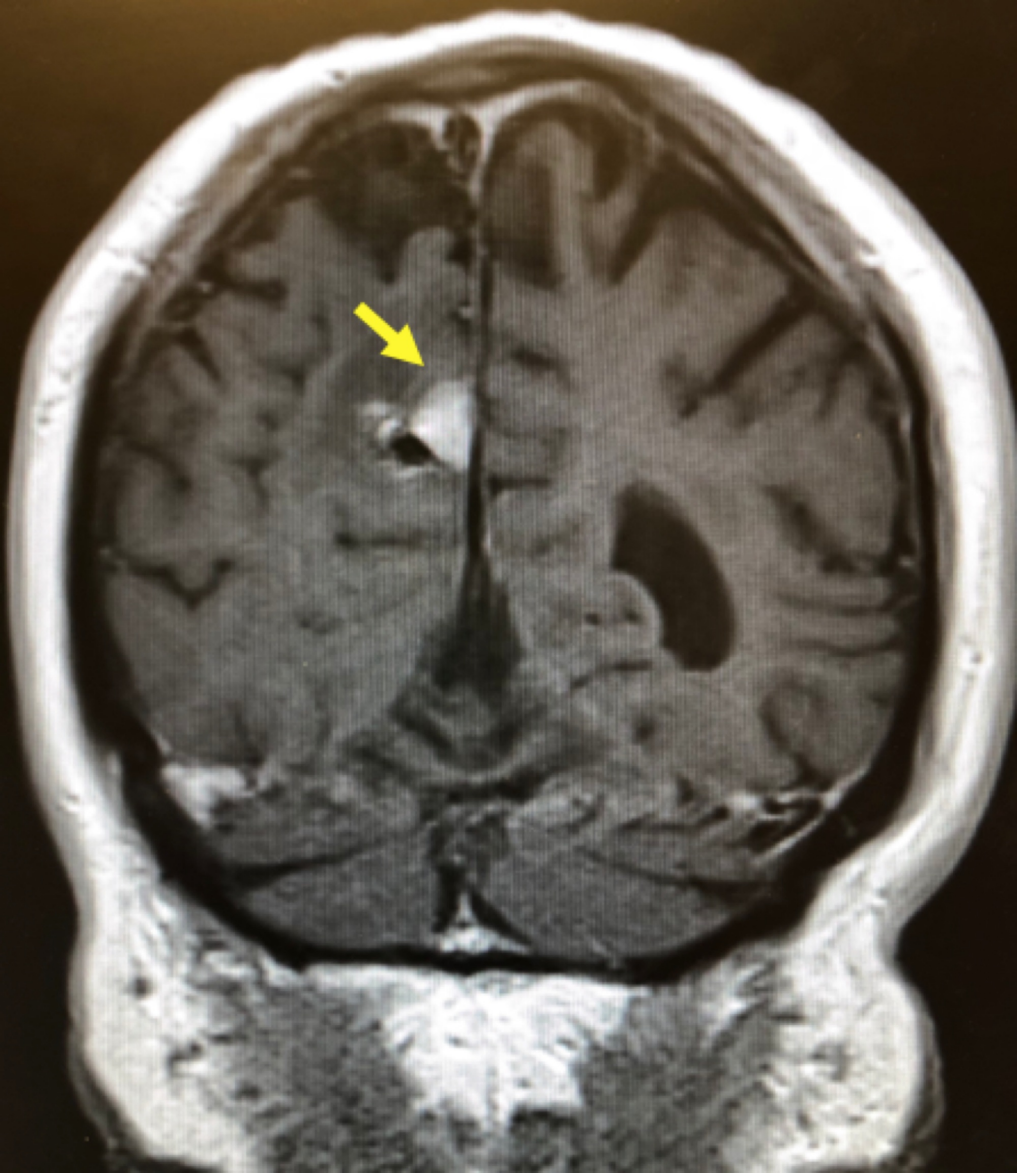
MRI brain with and without contrast demonstrating an enhancing lesion in the right frontal cortex 5 mm x 7 mm in diameter MRI: magnetic resonance imaging

**Figure 2:**
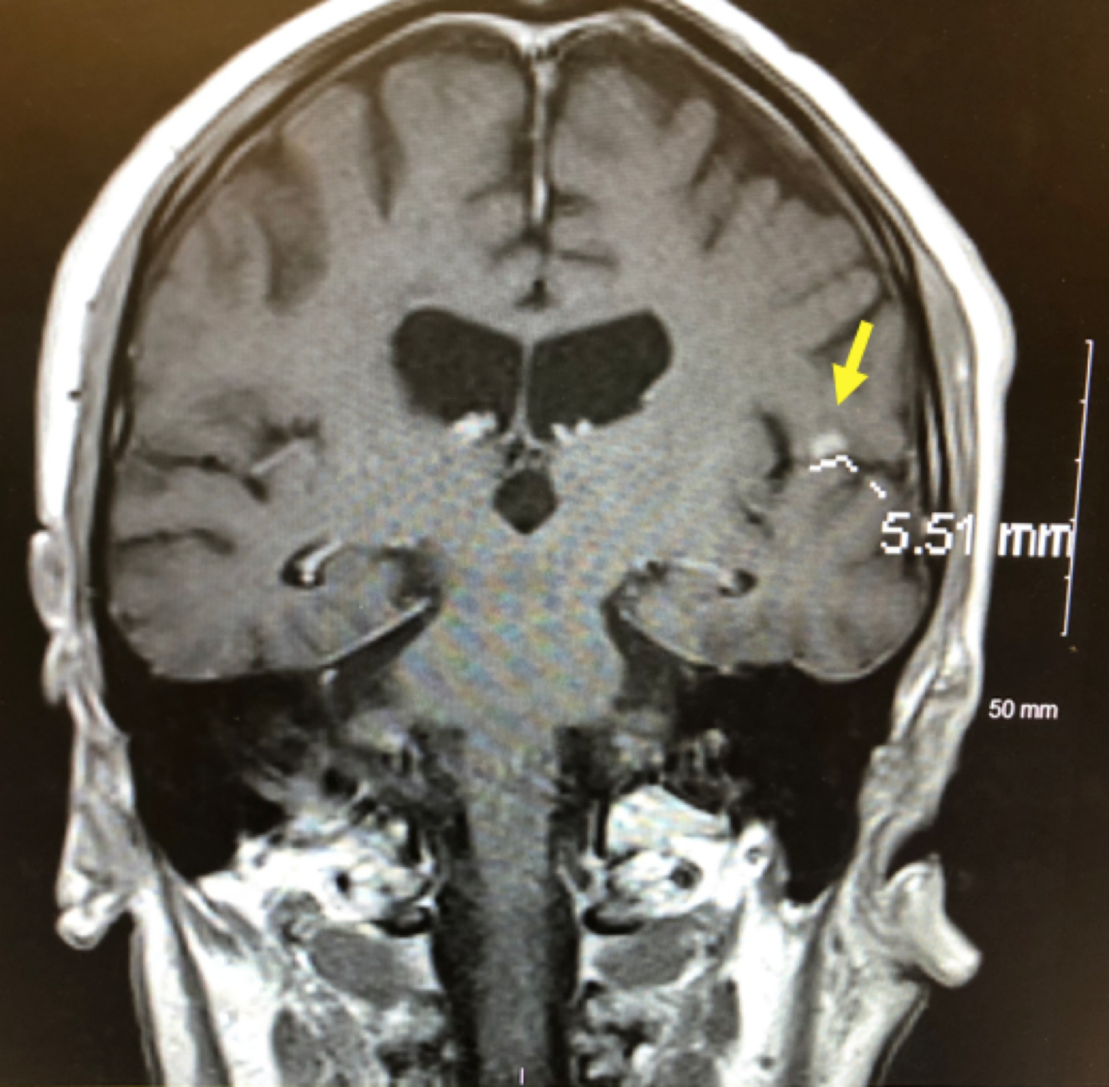
MRI brain with and without contrast demonstrating a 5.51 mm enhancing lesion in the left temporal operculum MRI: magnetic resonance imaging

**Figure 3:**
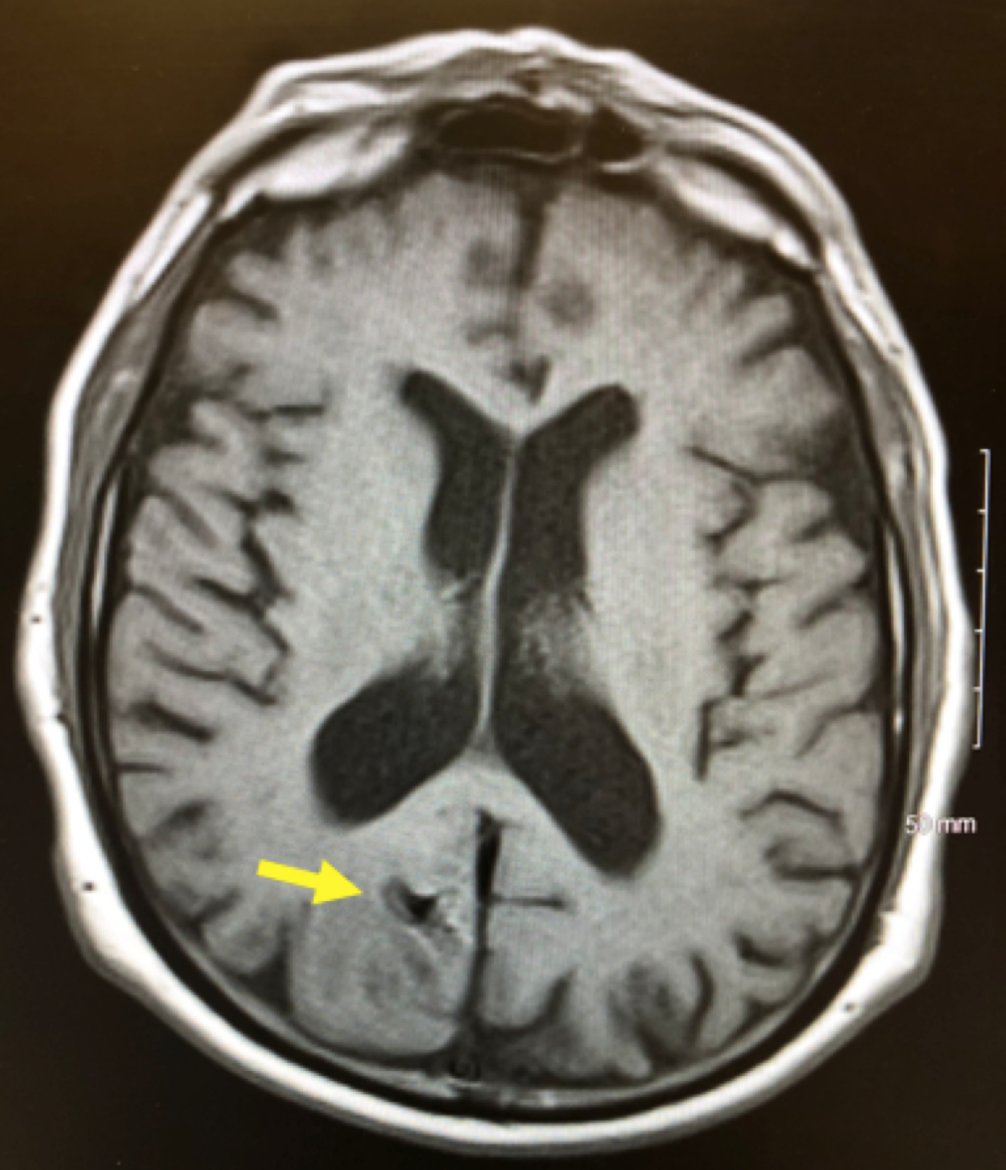
MRI brain with and without contrast demonstrating an enhancing mass in the right parieto-occipital medial cortex approximately 1.2 cm in diameter MRI: magnetic resonance imaging

**Figure 4:**
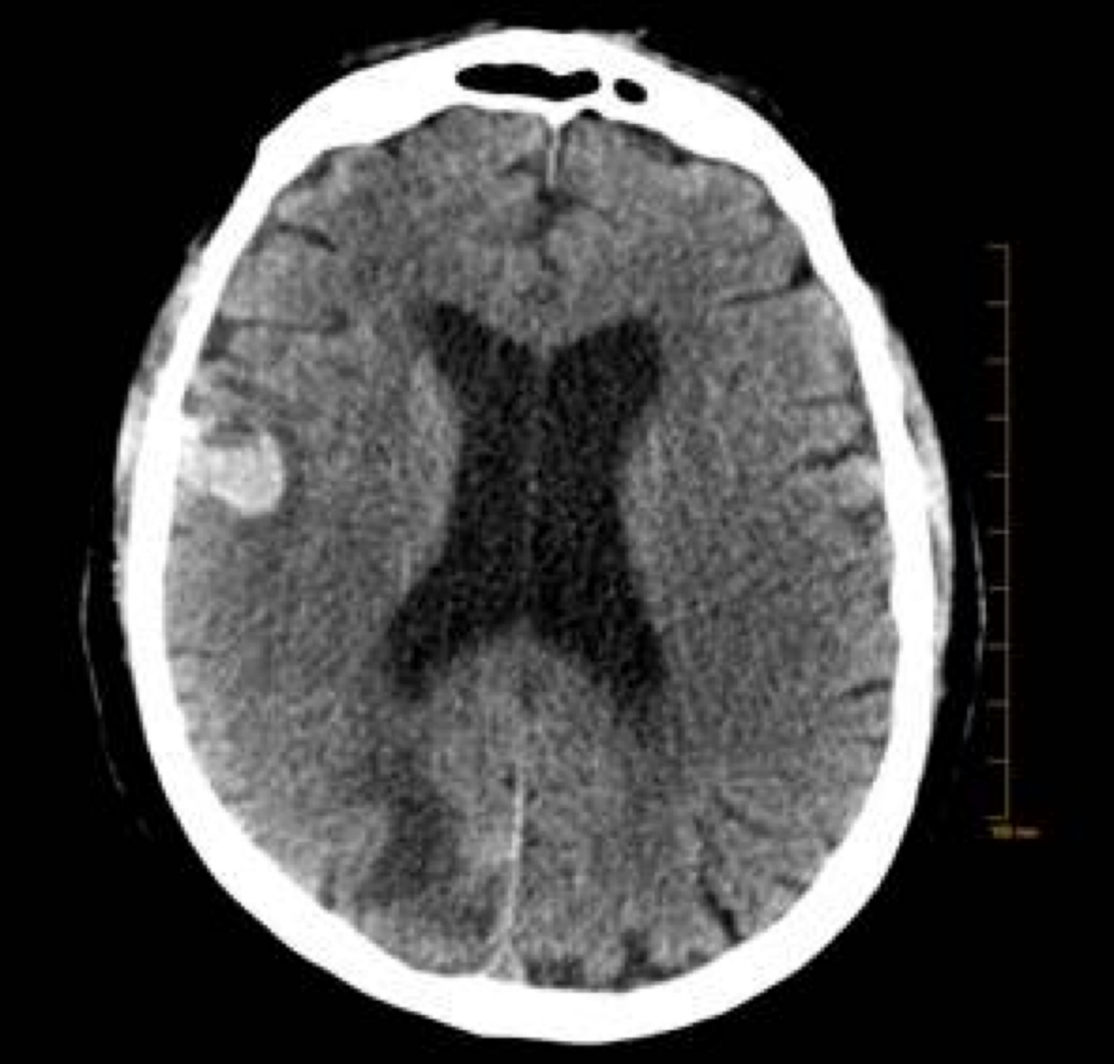
CT brain without contrast demonstrating a rounded area of hemorrhage with hyperdensity measuring 1.8 cm involving the right frontal cortex just above the Sylvian fissure with surrounding slight vasogenic edema CT: computed tomography

An 81-year-old Caucasian male with a diagnosis of melanoma on the right anterior chest wall and a wide local excision was found to have a recurrence in the right lung four months after initial diagnosis. Pembrolizumab was initiated, however, treatment was switched to dabrafenib and trametinib once molecular studies confirmed positivity for the BRAF V600R mutation. Nine months after diagnosis, magnetic resonance imaging (MRI) of the brain with and without contrast revealed multiple metastatic lesions in the brain (Figures 1-3), prompting the administration of the second cycle of pembrolizumab in addition to dabrafenib and trametinib. One month following the second cycle of pembrolizumab, the patient was admitted to the hospital with progressive weakness described as originating in the bilateral LEs and then spreading to his bilateral UEs. His neurological exam revealed a strength of 2/5 in the bilateral UEs and 0/5 in the bilateral LEs with areflexia and no bulbar muscle weakness. A lumbar puncture showed an albuminocytologic dissociation with elevated CSF protein (56 g/L), which supported our diagnosis of AIDP. Electromyography was consistent with motor and sensory neuropathy. Infectious workup revealed negative CSF cultures and VDRL. Antibodies to ganglioside GM1 for GBS were negative. He was promptly started on methylprednisolone and IVIG for five days. On Day 1 of treatment, he developed acute hypoxic respiratory failure, requiring mechanical ventilation. After five days of treatment without any clinical improvement, plasmapheresis was initiated. On Day 14 of hospitalization, he developed a generalized tonic-clonic seizure. A computed tomography (CT) scan of the brain without contrast revealed a hemorrhage within one of his metastatic lesions and associated vasogenic edema (Figure 4). On the same day, care was withdrawn and the patient expired shortly after.

## Discussion

IO therapy has shown a clinical benefit in several solid tumor types and in some hematological malignancies, improving either progression-free survival and/or overall survival. Currently, the use of IO therapies has moved from the metastatic setting into the early stages, and clinical trials are underway in the neoadjuvant and adjuvant scenarios. Approach to treatment is also moving from monotherapy to combined regimens, including molecularly targeted therapies, chemotherapy, and radiotherapy. Thus, we can expect an increase in the number of irAEs induced by IO that is more than what has been cited in the literature from various clinical trials. We can either block the PD-1 receptor (e.g. nivolumab and pembrolizumab) or bind the PD-L1 ligand by using an anti-PD-L1 monoclonal antibody (e.g. atezolizumab, durvalumab, or avelumab), avoiding the interaction between this specific ligand and its receptor. When the PD-1 checkpoint pathway is intact, the activity of T-cells in peripheral tissues during an immune response is decreased. This physiologic pathway limits autoimmunity, however, malignant tumors use it to evade host immune surveillance. By upregulating PD-L1 on their cell membrane, tumor cells promote the interaction of PD-L1 with PD-1 and B7.1 (CD80) receptors [3]. CD80 is another co-inhibitory receptor on the activated T-cell lymphocytes [3]. Hence, by targeting PD-1 receptors, pembrolizumab enhances the host antitumor immune response by effector T-cell lymphocytes [3,5].

By removing the inhibition over the activated T-cell lymphocytes, checkpoint inhibitors inadvertently remove self-tolerance, thereby predisposing patients to a unique profile of irAEs [2,4,7]. Many irAEs have been well-documented, including pneumonitis, colitis, hepatitis, nephritis, and endocrinopathies such as hypophysitis, thyroid disorders, adrenal insufficiency, and type 1 diabetes mellitus [4-5]. Neurologically related AEs are less understood and have been infrequently cited in the medical literature. Thus far, the incidence of grade 3 or 4 neurological irAEs reported by all IO approved for clinical use is less than 1% [10]. Zimmer et al. showed that irAEs occurred in 27.8% of patients taking anti-PD-1 therapy with nivolumab or pembrolizumab, with the most common adverse outcomes affecting the respiratory tract and musculoskeletal system [6]. In the same study, neurologic irAEs of all grades occurred in 3.2% of the patients [6]. Reported neurologic adverse effects include tremors, dysarthria, ataxia, paresis, and paresthesias [6]. Infrequent cases of aseptic meningitis, AIDP/GBS, transverse myelitis, myasthenia gravis, and enteric neuropathy have also been reported [6-7]. The only case of GBS observed in the aforementioned study was noted 15 weeks after initiation of treatment with nivolumab. This case was treated with methylprednisolone and IVIG, which led to the resolution of symptoms [6]. Other cases of peripheral neuropathy have been documented with variable presentations of sensory and motor dysfunction in the UEs and LEs. Kao et al. revealed four such cases with one case suspected to be AIDP with prominent facial diplegia and CSF findings of elevated protein and mild pleocytosis [7].

It has been hypothesized that melanocytes and Schwann cells share many antigens together, as they are both derived from neural crest cells, which suggests that an immune response against melanocyte antigens could also attack similar antigens of Schwann cells [8,11].^ ^This presents the possibility that the pathogenesis revolves around a cross-reaction between antibodies. In our patient cases, pembrolizumab was used for the treatment of metastatic melanoma and adenocarcinoma of the lung. Further studies are required to understand the mechanism of development of these AEs in not just melanoma but also with other indications for treatment with IO agents.

In patients who develop new-onset neurological symptoms after the initiation of checkpoint inhibitors (either PD-1 or PD-L1 antibodies), suspicion should be high for an irAE. The symptoms of progressive weakness, which was greater in the LEs than in the UEs, and the associated areflexia in our patients were highly suggestive of AIDP. Our diagnosis was further supported by CSF findings of albuminocytologic dissociation. Infectious workup was negative for both patients. Paraneoplastic syndromes were considered and ruled out in both cases. In case two, electromyography was suggestive of motor and sensory neuropathy. Our patients developed severe AIDP following the first course of treatment in case one and the second course of treatment in case two. Symptoms arose within three to four weeks of each respective dose. Both patients were treated with corticosteroids in addition to IVIG and plasmapheresis. A partial improvement of AIDP was observed in the first case. In the second case, AIDP was suspected to lead to respiratory failure, however, the hemorrhage of a metastatic brain lesion accelerated his clinical deterioration.

For patients on IO therapy, presenting with neurological symptoms, one should consider a quick evaluation with imaging studies (magnetic resonance imaging of the brain and/or spine), lumbar puncture, viral serologies, and cultures. Once the workup has been initiated and clinical suspicion is high for AIDP, further workup should include the paraneoplastic panel along with electromyography. The patient should be treated with high doses of intravenous corticosteroids (e.g. methylprednisolone). Other therapeutic interventions, such as IVIG and plasmapheresis, may be necessary if no clinical improvement is seen with steroids. The aim of these interventions is to suppress and decrease the autoimmune reactivity elicited by IO agents.

## Conclusions

Any new or worsening neurological symptom in a patient receiving an IO agent (regardless of the diagnosis) should prompt an immediate evaluation by neurology. Clinicians must be keen to recognize AIDP as a complication of IO therapy, as a misdiagnosis or delay in therapy could be fatal. Early intervention can potentially prevent complications such as respiratory failure and death. More research is needed to understand the development of these rare irAEs and attempt to ascertain risk factors, which may predispose cancer patients to these neurological syndromes.
